# Hand‐Driven Gyroscopic Hybrid Nanogenerator for Recharging Portable Devices

**DOI:** 10.1002/advs.201801054

**Published:** 2018-09-27

**Authors:** Jihoon Chung, Hyungseok Yong, Haksung Moon, Quang Van Duong, Seung Tae Choi, Dongseob Kim, Sangmin Lee

**Affiliations:** ^1^ School of Mechanical Engineering Chung‐Ang University 84 Heukseuk‐ro Dongjack‐gu Seoul 06974 Republic of Korea; ^2^ Aircraft System Technology Group Korea Institute of Industrial Technology (KITECH) 57 Yangho‐gil Yeongcheon‐si, Gyeongsangbuk‐do 38822 Republic of Korea

**Keywords:** energy harvesting, gyroscopic generators, hybrid generators, piezoelectric nanogenerators, triboelectric nanogenerators

## Abstract

With the rise of portable and wearable electronics, a fast‐charging, long‐lasting power solution is needed; thus, there are attempts to harvest energy from the ambient environment. Mechanical energy harvesting through piezoelectric and triboelectric nanogenerators (PENG and TENG) is a promising approach due to their light weight, low cost, and high‐power density in comparison to other technologies. Both types of generators are capable of charging portable and smart devices on their own by converting mechanical energy into electricity. However, most previous methods have excessive input conditions, such as high rpm and input frequency, that can be only applied with other actuators. Here, a hand‐held gyroscopic generator is presented that uses the gyroscopic principle to reach a rotation rate above 8000 rpm with only hand input. The generator comprises a rotating flywheel inside a casing. Both the flywheel and casing have a TENG, and with a hybrid generator, electrical power is produced from rotation, vibration, and centrifugal force during operation. The device shows a consistent open‐circuit voltage (*V*_OC_) of 90 V and a closed‐circuit current (*I*_CC_) of 11 µA with a frequency of 200 Hz. As a stand‐alone device, this generator can power portable sensors and smartphones through hand rotation.

## 1 Introduction

In past decade, there has been great interest in developing a fast‐charging and long‐operating power solution as portable devices have rapidly spread and interest in wearable electronics has risen. Although there have been various studies on batteries and charging methods, solutions for frequent recharging and powering of portable and wearable electronics are still required. To satisfy this need, there have been attempts to develop stand‐alone power generators that can produce electricity from ambient energy sources such as solar,^1^ mechanical,^2^ and thermal energy.^3^ Among these approaches, mechanical energy harvesting is a promising method that can generate electrical power regardless of external environment conditions, such as weather and temperature. The piezoelectric nanogenerator (PENG) is a mechanical energy harvesting device that utilizes piezoelectric material to harvest kinetic energy into electricity,^4^, ^5^ and recently the triboelectric nanogenerator (TENG), which utilizes contact electrification, has been introduced.^6^, ^7^, ^8^ These methods have attracted great interest due to their light weight, low cost, and high‐power density in comparison to other existing energy harvesting technologies.^9^, ^10^, ^11^ Previous studies have demonstrated that both PENGs and TENGs are capable of powering portable devices on their own by converting mechanical energy into useful electricity.^10^, ^11^, ^12^, ^13^ However, most previous methods of powering portable electronics have excessive input conditions. The input conditions of previous works can only be applied with certain actuators, such as high pushing force of 50 N (5 kgf),^14^ rotation speed over 400 rpm,^10^, ^15^ and frequency of 30 Hz.^16^ Further, this could cause serious wear and abrasion of materials, consequently leading to a short life span. For mechanical energy harvesters to be widely used in portable electronics, the input energy should be limited to human power. Therefore, the development of a small, portable energy harvester is needed that can generate considerable output with high frequency to charge batteries or power electronics on its own.

In this paper, we introduce a hand‐driven gyroscopic generator that can harvest rotating, vibrating mechanical energy into electricity. This generator has the shape of a gyroscopic wrist exerciser; it consists of a rotating flywheel held inside an external casing. Due to the gyroscopic motion, the flywheel can reach above 8000 rpm with only hand motion which can significantly increase the output frequency and power. We fabricated a TENG on the flywheel surface and a triboelectric–piezoelectric hybrid generator on the casing substrate; thus, the gyroscopic generator can generate electrical power from all mechanical energy during operation. In addition, the flywheel TENG has an alternating pattern of triboelectric materials with different electron affinities, which can increase the electrical power output of the freestanding‐type TENG by 19%. On the outer surface of the casing, a poled poly(vinylidene fluoride‐co‐trifluoroethylene) (P(VDF‐TrFE))‐based TENG and PENG hybrid nanogenerator is fabricated to harvest energy from hand gripping, centrifugal force, and vibration. The gyroscopic generator has shown a consistent open‐circuit voltage (*V*_OC_) of 90 V and a closed‐circuit current (*I*_CC_) of 11 µA with a frequency of 200 Hz. To demonstrate the feasibility of the proposed generator as a stand‐alone device, it was used to power a portable clock and smartphone through hand rotation.

## 2 Results and Discussion

**Figure 1:**
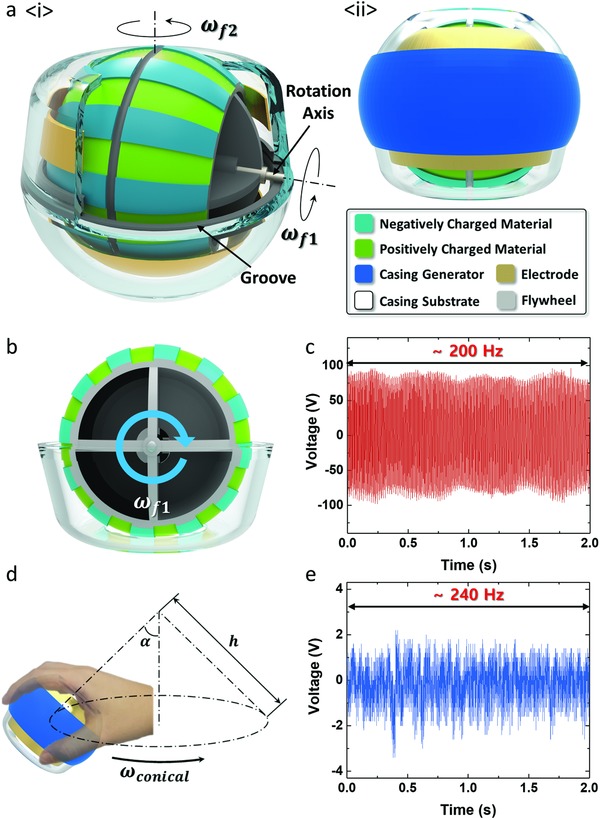
Hand‐driven gyroscopic generator. a) Schematic of gyroscopic generator (i), and casing generator (ii). b) Rotation of flywheel inside the casing substrate. c) *V*_OC_ of flywheel TENG. d) Rotating gyroscopic generator by hand. e) *V*_OC_ of casing generator.

The gyroscopic generator has a flywheel rotating in the casing substrate as shown in **Figure** **1**a(i). As the operator rotates the gyroscopic generator, the flywheel rotates in the direction of ω_f2_ and rotation axis roll against the groove in direction of ω_f2_ due to the friction force between the rotation axis and the groove. By the gyroscopic movement, rotation speed of flywheel can reach over 8000 rpm just by hand power. This high‐speed rotating energy of the flywheel can be harvested through the TENG located inside. To effectively collect rotational energy, flywheel has an alternating pattern of negatively charged and positively charged materials around its surface. The positively charged material used in this study is polyurethane film, and the negatively charged material is polytetrafluoroethylene (PTFE) film. 1 cm wide copper sheets are adhered to the inner surface of the upper and lower casing substrate as electrodes to harvest the rotating energy of the patterned flywheel. The whole gyroscopic generator is approximately the size of a tennis ball, so the operator can grasp and rotate it easily. As shown as Figure 1a(ii), the casing generator, which has a triboelectric and piezoelectric hybrid nanogenerator inside, is fabricated along the outside surface of the casing substrate at the position where the operator grasps the gyroscopic generator. This casing generator has poled P(VDF‐TrFE) inside, which is a piezoelectric material that can be utilized in TENGs as well. The casing generator produces electrical energy when the operator first grasps the gyroscopic generator; a pressure difference occurs due to centrifugal force, and vibration is produced by flywheel rotation. A detailed explanation of the casing generator and the experiment conducted on it is provided in Figure 3. Through the TENG fabricated on the flywheel and the casing generator, the gyroscopic generator can harvest all types of mechanical energy during operation.

The rotation and acceleration of the flywheel can be explained by gyroscopic precession. As shown in Figure 1b, the main direction of rotation that affects the output of the flywheel TENG is the direction of angular velocity ω_f1_. The flywheel rotates with two degrees of freedom, one rotating in the *x*‐axis with angular velocity of ω_f1_, and the other in the *y*‐axis with angular velocity of ω_f2_ (Figure S1a, Supporting Information). On the inner side of the casing substrate, there is a groove where the rotating axis of the flywheel (*x*‐axis) can be positioned and can rotate in direction of ω_f2_. The groove size is slightly bigger than the flywheel axis, so the flywheel can be slightly tilted (Figure S1b, Supporting Information). The casing of the gyroscopic generator is tilted during rotation by hand. Thus, one end of the flywheel axis is in contact with the upper surface of the groove, and the other end is in contact with the bottom surface when the flywheel is spinning. This makes the rotating flywheel receive torque to rotate in the direction of ω_f1_ when rotation is conducted by hand.

When the gyroscopic generator is rotated by hand, the flywheel axis is pressed against the groove surface due to gyroscopic precession, and this provides torque to the rotating flywheel. As the speed of hand rotation increases, the flywheel tends to tilt more due to the increased precession velocity, and the force between the groove and the flywheel axis increases as well. This increases the torque, and the flywheel axis starts to move along the groove (*v*_1_, Figure S1c, Supporting Information), and friction force makes ω_f1_ faster. When the speed of hand rotation is constant, the precession velocity decreases as the flywheel rotation in the *y*‐axis (ω_f2_) and the *x*‐axis (ω_f1_) increases. At this stage, the flywheel reaches dynamic equilibrium, where the axis of the flywheel rotates in the ω_f2_ direction without slipping.^17^ To increase the rotation of the flywheel (ω_f1_), the operator must increase the speed of rotation by hand.^18^ The open‐circuit voltage (*V*_OC_) of the flywheel TENG is shown in Figure 1c when the gyroscopic generator is rotated by hand. As shown in the plot, the peak *V*_OC_ is measured as 90 V, and the frequency of the waveform is 200 Hz. The voltage has high frequency (Hz) due to the high‐speed rotation of the flywheel, which is over 8000 rpm. The rotation rate of flywheel during operation is measured by rpm meter connected to the gyroscopic device in Figure S2 of the Supporting Information.

Figure 1d is a schematic of hand motion rotating the gyroscopic generator. When the operator increases the rotation of the flywheel, the gyroscopic generator is rotated in a conical shape with the precession angle of α and the length of *h*. The flywheel must rotate at a certain speed before rotating the generator in the ω_conical_ direction. If the flywheel is stationary, the flywheel does not accelerate, even if the gyroscopic generator is rotated. As previously mentioned, once the flywheel is rotating at sufficient speed in the ω_f1_ direction and precession rotation is given, the speed of the rotating flywheel increases. During rotation, the flywheel vibrates inside the casing cylinder, and the centrifugal force applied to the gyroscopic generator constantly changes. Due to this mechanical energy, the casing generator on the surface of the casing substrate generates electrical output. The *V*_OC_ output produced by the casing generator during operation is shown in Figure 1e. The casing generator can produce a peak voltage of 2.2 V at 240 Hz.

**Figure 2:**
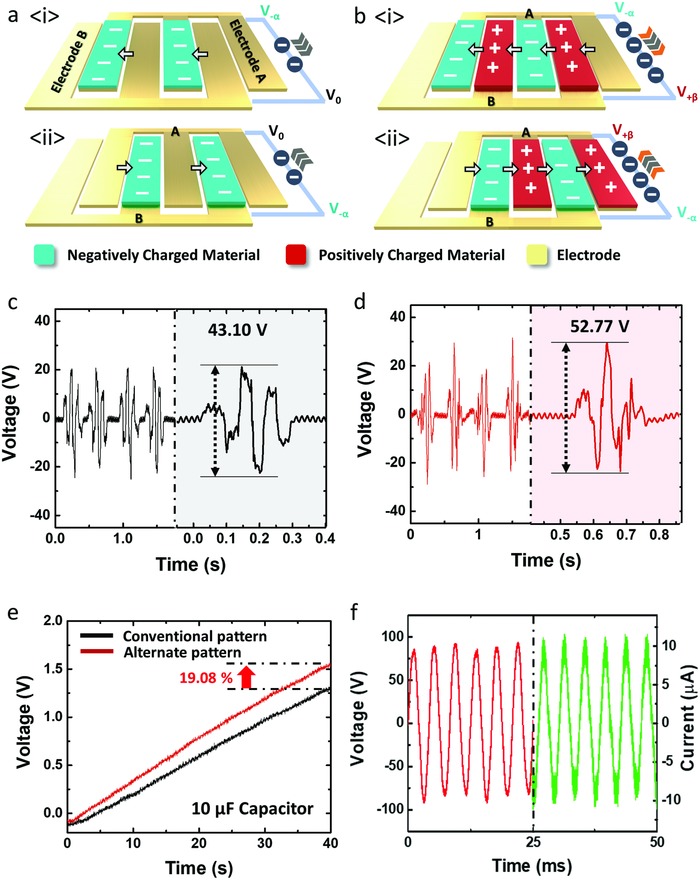
Alternating pattern TENG. Schematics of a) conventional pattern TENG and b) alternating pattern TENG. *V*_OC_ output of c) conventional pattern TENG and d) alternating pattern TENG. e) Capacitor charging rate of conventional pattern and alternating pattern TENG. f) *V*_OC_ and *I*_CC_ output of flywheel TENG with alternating pattern.

As shown in Figure 1a, the flywheel TENG has an alternating pattern around the flywheel surface to effectively harvest rotation energy when the flywheel is rotating at high speed. The electrode on the inner surface of the casing substrate is stationary, while the pattern on the flywheel is constantly rotating. This generation mode of the TENG is called freestanding mode.^19^, ^20^ **Figure** **2**a is a schematic of a typical grating pattern used in conventional pattern freestanding‐mode TENGs. As shown in the schematic figure, there are triboelectric materials on the electrode surface. Both electrodes (electrodes A and B) are stationary while triboelectric material is moving back and forth due to external force. First, when the negatively charged material comes into contact with electrode A (Figure 2a(i)), the electrical potential of electrode A becomes proportional to the magnitude of the surface charge density of the negatively charged material (*V_−_*_α_), and the electrical potential of electrode B becomes the initial state (*V*_0_).^21^ Due to the electrical potential difference between the two electrodes, the electrons of electrode A are transferred to electrode B to maintain an electrical equilibrium state (*V_−_*_α_ *– V*_0_). When the negatively charged material moves from electrode A to electrode B (Figure 2a(ii)), the electrical potential difference between A and B is reversed, and electrons flow in the opposite direction. In this case, the magnitude of voltage and current generated between A and B is mostly determined by the negatively charged material. To increase the electrical potential difference, in many previous freestanding‐mode TENGs, the substrate material has been removed between the pattern materials to create empty spaces^10^, ^22^, ^23^ because most substrate polymers are more likely to be negatively charged than metal acting as an electrode. However, this method is difficult to apply in devices that have fully packaged designs. To overcome this drawback, we designed an intuitive and simple pattern that could be applied to any structure, and it achieved better performance than conventional patterns.

Figure 2b shows the alternating pattern design of the TENG for a flat substrate without any empty space. As seen in Figure 2b, the alternate pattern of positively and negatively charged material is on electrodes A and B moving back and forth. The surface charge of the positively charged material used in this study is higher than the positive surface charge of metal electrodes. As mentioned previously, electrode A has the electrical potential of *V_−_*_α_ due to the negatively charged material. However, electrode B has the electrical potential of *V*_+β_ affected by the positively charged material. Compared to Figure 2a, the electrical potential difference between electrodes A and B can be expressed as *V*_−α_ *– V*_+β_ when two triboelectric materials are alternately patterned. This enhances the electrical potential difference between the two electrodes and transfers a large number of electrons as external force is applied (Figure 2b(i,ii)).

Figure 2c,d shows the measured *V*_OC_ results of a conventional pattern and the alternating pattern. Aluminum, PTFE, and polyurethane were used in experiments as the electrode, negatively charged material, and positively charged material, respectively. As seen in the two plots, both patterns effectively generate alternative current according to the movement of the materials. However, the output of the conventional pattern is about 43.1 V, whereas the output of the alternating pattern is about 52.8 V, which is about 20% higher. To clearly compare the performance difference between the patterns, the charging rate of a 10 µF capacitor was measured (Figure 2e). Similar to the previous results, the alternating pattern showed a 19% faster charge rate than the conventional pattern. This alternate pattern can improve the performance of freestanding‐mode TENGs by a simple method that can be applied to various freestanding‐mode TENG devices. When this alternating pattern was applied to the flywheel TENG, the flywheel was designed to have empty space between the alternating patterns and the electrode. This is to avoid wear during flywheel rotation as it rotates in high speed. The working mechanism of flywheel TENG is as same as the alternating pattern TENG in Figure 2a. The alternating pattern is formed with PTFE and polyurethane on the flywheel surface. As the flywheel rotate, two materials pass alternately above 2 electrodes adhered on casing substrate of gyroscopic generator. The detailed working mechanism for flywheel TENG is shown in Figure S3 of the Supporting Information. The flywheel TENG showed a *V*_OC_ of 90 V and an *I*_CC_ of 11 µA at 200 Hz (Figure 2f).

**Figure 3:**
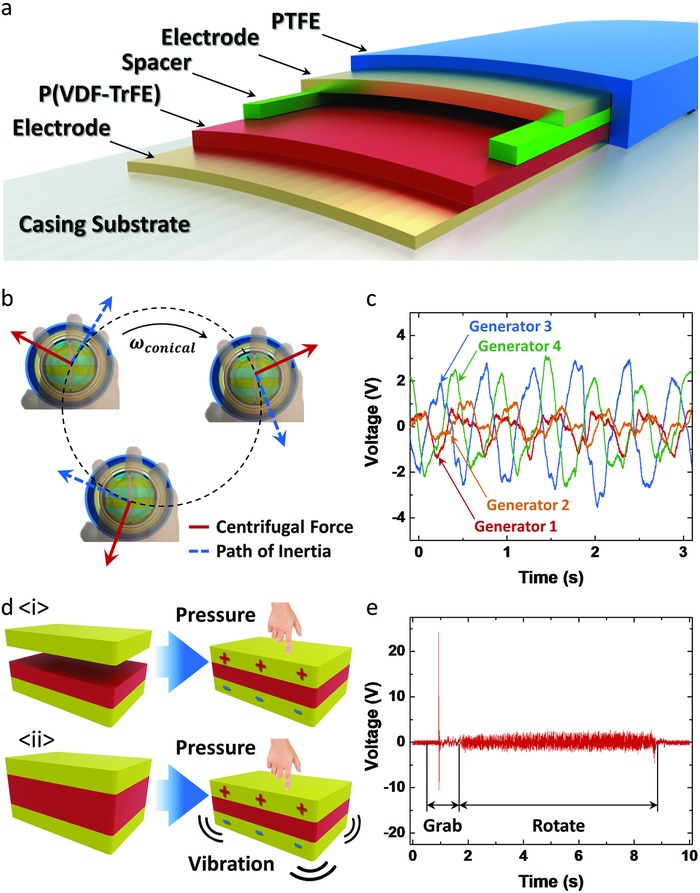
Casing generator. a) Cross‐sectional schematics of casing generator. b) Rotation of gyroscopic generator. c) *V*_OC_ output of casing generator due to centrifugal force. d) Triboelectric and piezoelectric mechanism of P(VDF‐TrFE). e) *V*_OC_ of casing generator output from grasping and rotation.

As the vibration of the flywheel and constant change of centrifugal force is applied to the gyroscopic generator, there is a need for a new design to collect these mechanical energies. **Figure** **3**a shows schematic drawings of the casing generator. Around the casing substrate of the gyroscopic generator, a sandwich composite of electrode‐P(VDF‐TrFE)–spacer–electrode–PTFE was adhered to the surface. A poled P(VDF‐TrFE) has a piezoelectric characteristic, which can harvest external pressure or vibration. A spacer is placed between the upper electrode and poled P(VDF‐TrFE) film so it can also generate output by triboelectric effect. 100 µm thick polyimide film is adhered with commercial double‐sided tape in between poled P(VDF‐TrFE) and upper electrode in this study. As shown in Figure S4a of the Supporting Information, the top and the bottom electrode can have an electrical potential difference from both the triboelectric effect and piezoelectric effect when it is pressed.^24^, ^25^ When the operator first presses the casing generator, the upper electrode comes into contact with the P(VDF‐TrFE) surface, and an electrical potential difference is created due to the surface charge. When the operator applies higher pressure without separation, an electrical potential difference is created by the piezoelectric effect. Therefore, electrons flow to balance the electrical potential difference between the two electrodes. In addition, on the outside of the casing generator, a thin PTFE layer is adhered to generate electrical output when the operator first grasps the gyroscopic generator or changes the grip during operation.^26^

Figure 3b is the path of motion when the operator rotates the gyroscopic generator to accelerate the flywheel. During rotation, centrifugal force is applied to the gyroscopic generator trying to escape from the orbit. The operator's fingers that are grasping the generator block the gyroscopic generator to stay in the orbit, and the casing generator is compressed by the operator's fingers and the casing of the gyroscopic generator. As the direction of centrifugal force changes during rotation, the compressed position of the casing generator also changes in relation to the direction of the force. To effectively observe the generated output of the casing generator, four generators are installed on the surface of casing cylinder where the pressing fingers are placed (Figure S4b, Supporting Information). As shown in Figure 3c, each generator creates an alternative current as the direction of centrifugal force changes. The phase difference between each of the four generators is about the same as the rotation continues. The output frequency is about 2 Hz, which is the same as the rotation speed of a hand‐rotating gyroscopic generator.

As shown in Figure 3d, the casing generator can harvest energy from pressure and vibration. Due to the spacer unit, there is a hollow space between the P(VDF‐TrFE) and the electrode. When pressure is applied, the casing generator can produce output due to the P(VDF‐TrFE) tribocharged surface in contact with the electrode (Figure 3d(i)). In a traditional TENG, two electrodes do not generate an electrical potential difference when the surface of the triboelectric material and the electrode stay in contact. However, poled P(VDF‐TrFE) is a strong piezoelectric material that can generate an electrical potential difference when pressure is applied by mechanical deformation. Therefore, even when the surface of P(VDF‐TrFE) and the upper electrode stay in contact, an electrical potential difference can be generated through external pressure or vibration (Figure d(ii)). Figure S5 of the Supporting Information shows the *V*_OC_ output when P(VDF‐TrFE) was tapped against the electrode and bent with the electrode. This result shows that P(VDF‐TrFE) can be used as both a triboelectric and piezoelectric material.

Figure 3e shows the *V*_OC_ output of the long casing generator covering the casing cylinder. The first high voltage peak is generated when the operator grasps the gyroscopic generator for the first time. Then when the casing generator starts to rotate, high‐frequency voltage output is generated from the vibration of the flywheel during rotation. The *I*_CC_ output of the casing generator and magnified graphs of *V*_OC_ and *I*_CC_ are shown in Figure S6 of the Supporting Information. The output frequency of the casing generator during operation is about 240 Hz, which is similar to the output frequency of the flywheel TENG. This indicates that the output of the casing generator is from the vibration of flywheel rotation.

**Figure 4:**
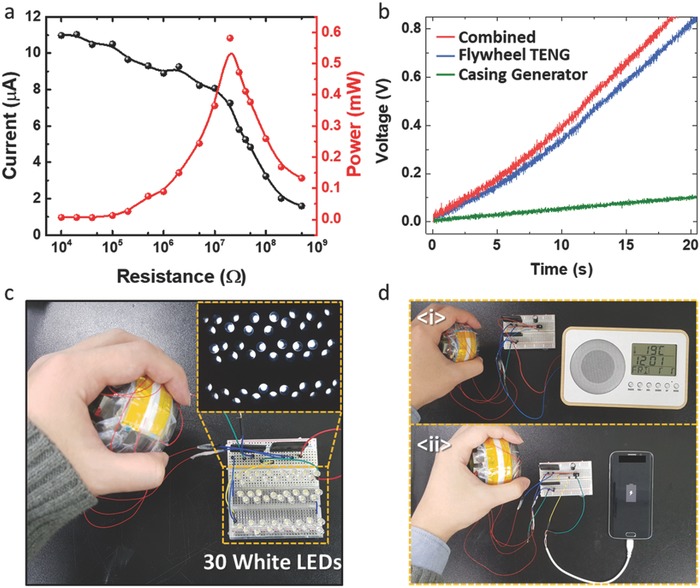
Output power and application of gyroscopic generator. a) Current and power output in relation to external loads. b) Capacitor charging rate of gyroscopic generator. c) Gyroscopic generator lighting 30 white LEDs. d) Gyroscopic generator powering a portable device (i), and smartphone (ii).

The peak electrical power produced by the gyroscopic generator is shown in **Figure** **4**a. The plot represents the current output in relation to the external resistance. As seen in the plot, the maximum peak power produced by the gyroscopic generator is about 0.581 mW at 20 MΩ. The voltage and current output in relation to the external resistance is illustrated in Figure S7 of the Supporting Information. In addition, a 220 µF capacitor was charged with the gyroscopic generator (Figure 4b). Both the flywheel TENG and the casing generator were connected to the capacitor using a rectifier circuit to increase the charging speed. With the combined power generation, it could charge the capacitor to 0.8 V in about 17 s when the generator was operated by hand.

To demonstrate the possibility of gyroscopic generator as auxiliary power source, the flywheel TENG and casing generator were connected to a rectifier circuit separately and shown to light up 30 white LEDs instantaneously with hand input (Figure 4c; Video S1, Supporting Information). In addition, as shown as Figure 4d, the gyroscopic generator was connected to a 1000 µF capacitor, and the power stored was then discharged by a portable application. In Figure 4d(i), the gyroscopic generator powered a portable device with a digital clock thermometer on an LCD screen (Video S2, Supporting Information). With the output of the gyroscopic generator, the portable device was powered constantly. The gyroscopic generator was also connected to a commercial smartphone (Figure 4d(ii)). The gyroscopic generator was able to charge the smartphone with the rectifier and capacitor combined circuit. Although the charging process was quite slow, the proposed generator could charge a commercial portable device with only hand rotation.

## 3 Conclusion

In summary, we successfully developed a gyroscopic generator that can generate high‐frequency electrical power through hand input. The gyroscopic generator uses the principle of gyroscopic precession, which induces high‐speed angular velocity of the flywheel inside it. The gyroscopic generator consists of two generator units: a flywheel TENG and a casing generator. In the flywheel TENG, an alternating pattern of positively and negatively charged material was adhered to the flywheel surface to harvest energy from high‐speed rotation of flywheel. The casing generator was made with poled P(VDF‐TrFE) between two electrodes to generate output from the surface charge and piezoelectric property. Due to its design, the casing generator can produce output through the triboelectric and piezoelectric effects. Through both generator units, this generator can produce electrical power from all motions of the device caused by rotation, vibration, and centrifugal force. Through flywheel rotation speed over 8000 rpm, the gyroscopic generator can produce a *V*_OC_ of 90 V and an *I*_CC_ of 11 µA with a frequency of 200 Hz. In addition, we demonstrated the potential of the gyroscopic generator as an auxiliary power source for portable electronics by powering portable electronics, including a smartphone. We believe that the gyroscopic generator developed in this study is a potential solution for powering portable electronics in the near future.

## Conflict of Interest

The authors declare no conflict of interest.

## Supporting information

Supplementary

Supplementary

Supplementary
