# Supplementary material for: Hand‐Driven Gyroscopic Hybrid Nanogenerator for Recharging Portable Devices
Source: Adv Sci (Weinh). 2018 Sep 27;5(11):1801054. doi: 10.1002/advs.201801054 (PMC6247056; doi:10.1002/advs.201801054)
Supplement: Supplementary file 1 — Supplementary [file ADVS-5-1801054-s002.pdf]

Copyright WILEY-VCH Verlag GmbH & Co. KGaA, 69469 Weinheim, Germany, 2018.

ADVANCED SCIENCE
Open Access

Supporting Information

for *Adv. Sci.,* DOI: 10.1002/advs.201801054

Hand-Driven Gyroscopic Hybrid Nanogenerator for Recharging Portable Devices

Jihoon Chung, Hyungseok Yong, Haksung Moon, Van Duong Quang, Seungtae Choi, Dongseob Kim, and Sangmin Lee**

Supporting Information

Hand-driven Gyroscopic Hybrid Nanogenerator for Recharging Portable Devices

*Jihoon Chung, Hyungseok Yong, Haksung Moon, Quang Van Duong, Seungtae Choi, Dongseob Kim, and Sangmin Lee**

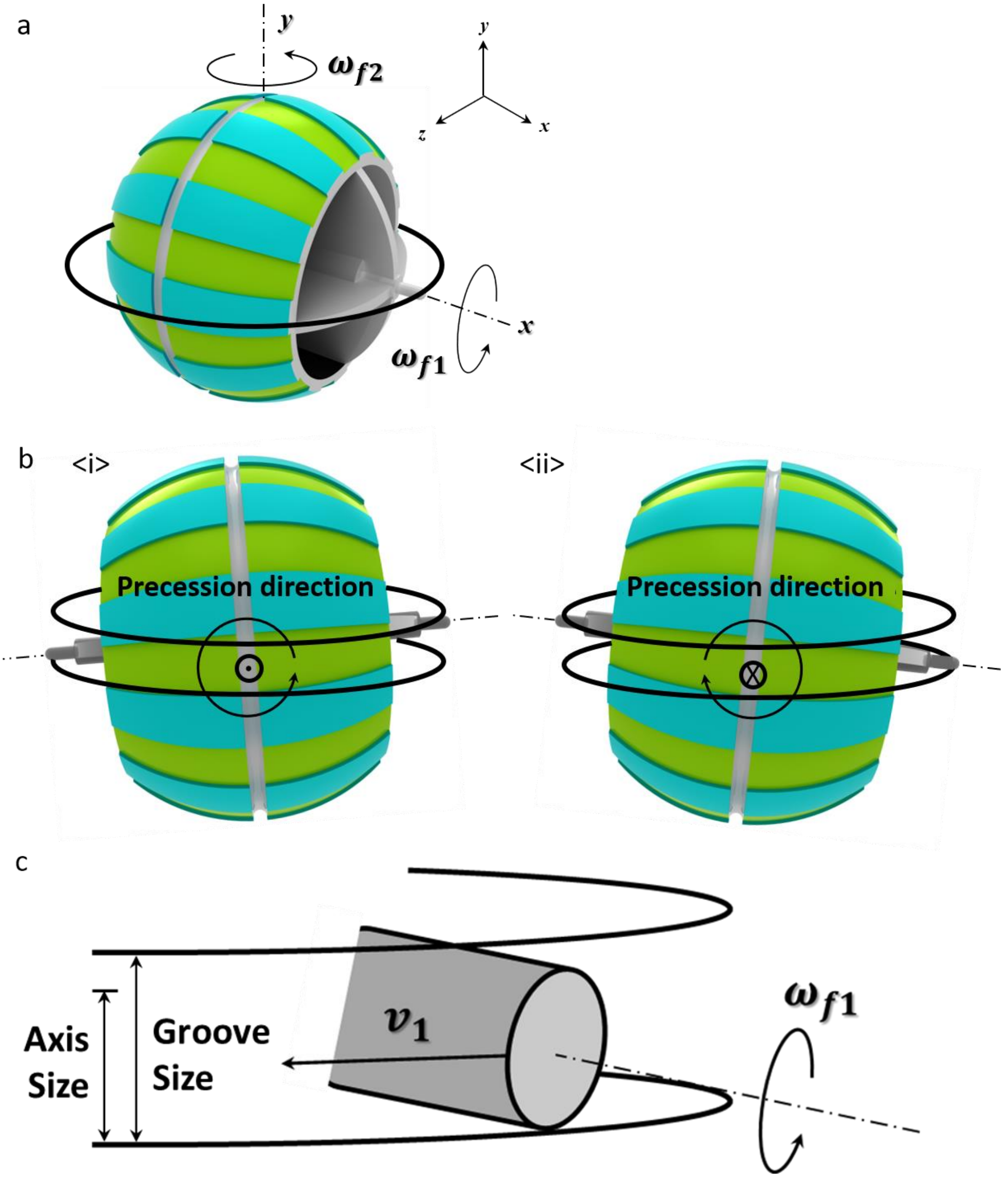

Figure S1. Markers for gyroscopic motion. (a) Markers for flywheel rotation. (b) Tilted flywheel due to groove. (c) Torque applied to axis of flywheel.

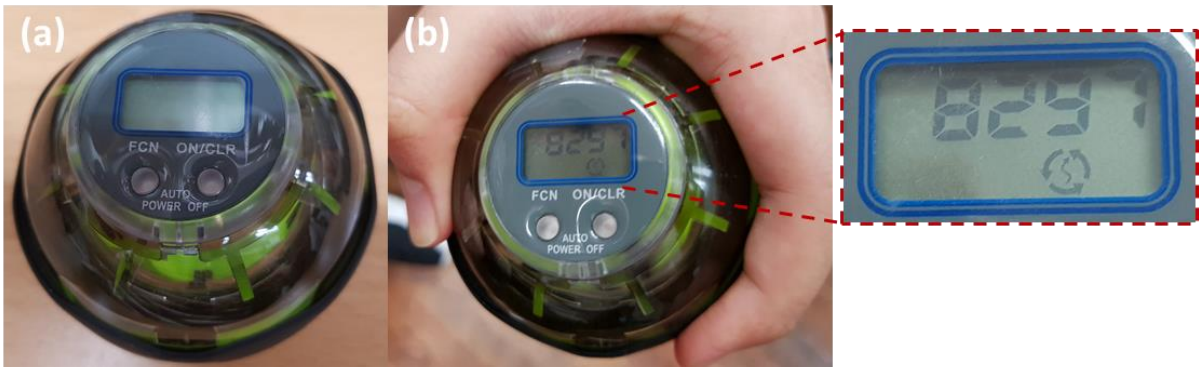

Figure S2. RPM meter readings during gyroscopic generator operation (8297 rpm). (a) RPM sensor on top of gyroscopic generator. (b) RPM reading during operation.

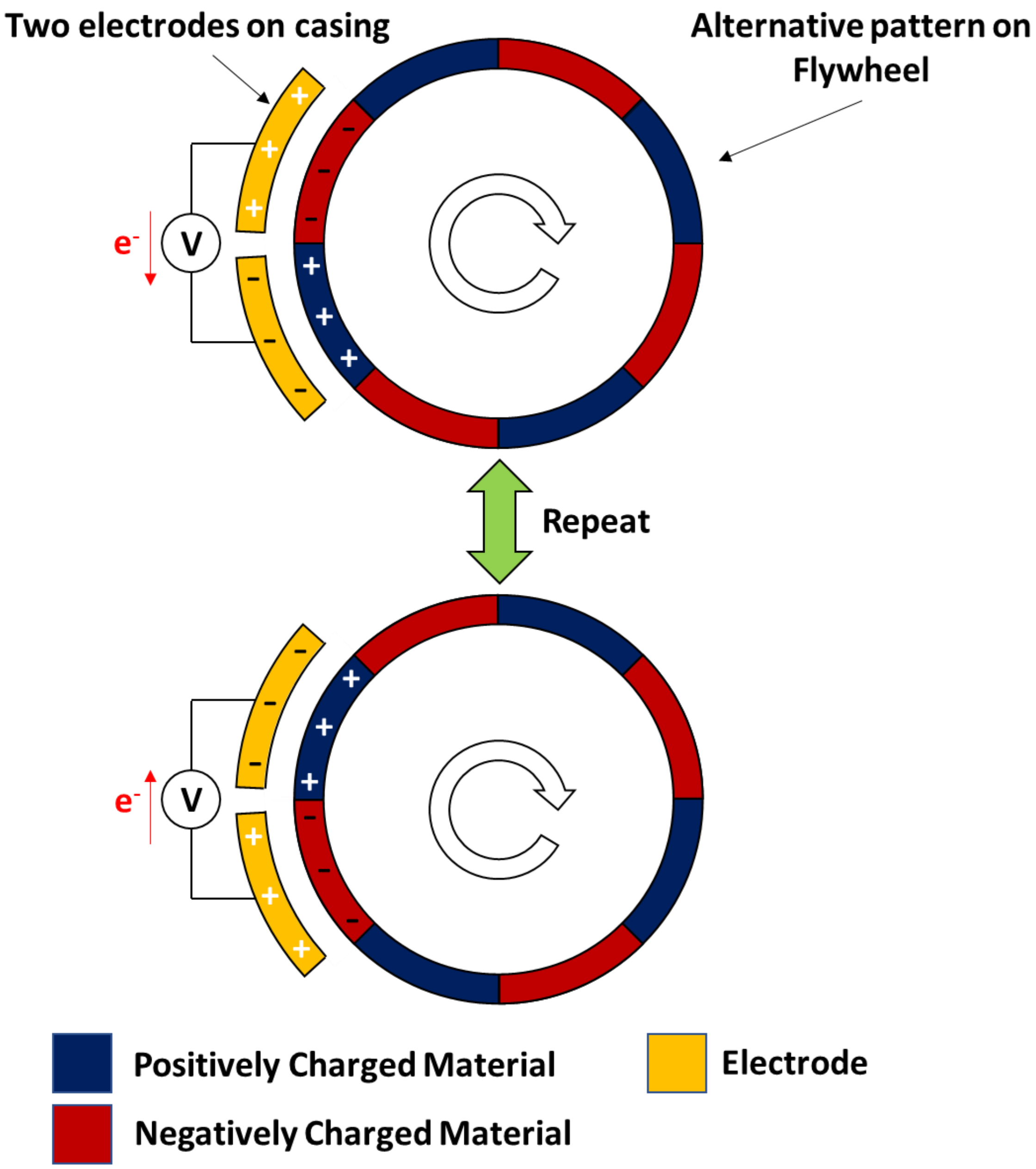

Figure S3. Working mechanism of flywheel TENG.

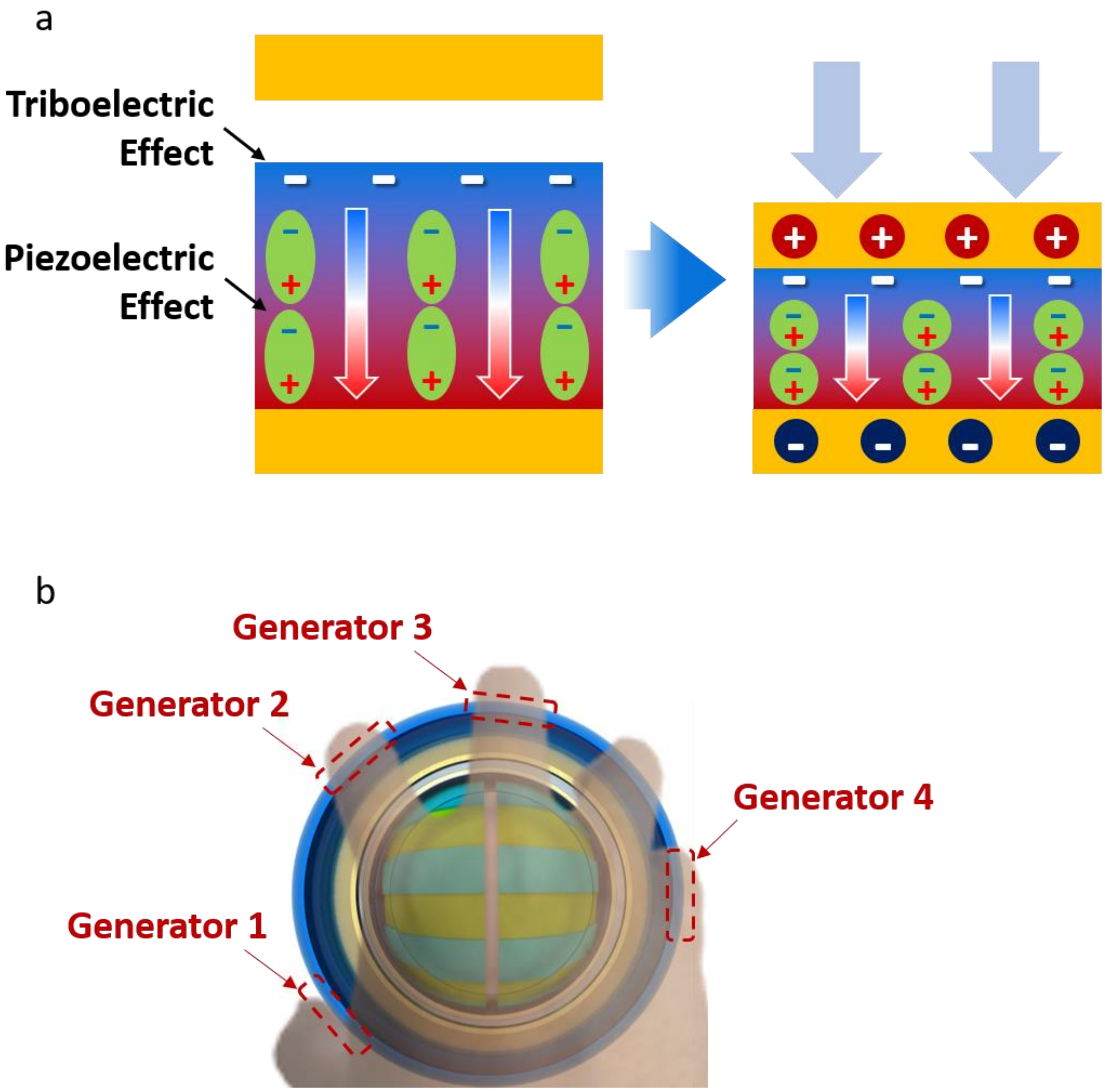

Figure S4. Mechanism and position of casing generator. (a) Casing generator using both triboelectric effect and piezoelectric effect. (b) Positions of 4 casing generators in Figure 3c.

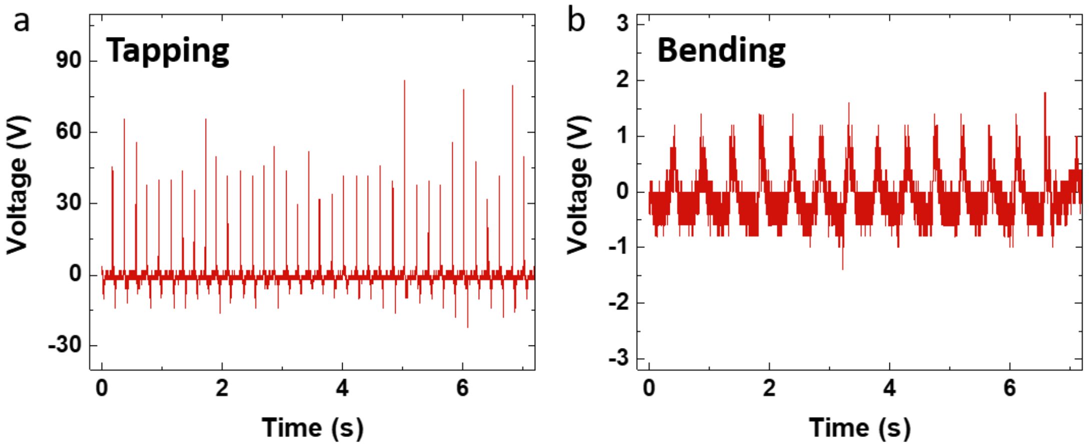

Figure S5. Triboelectric and piezoelectric property of P(VDF-TrFE). V_{OC} output of P(VDF-TrFE) generator during (a) tapping and (b) bending motion.

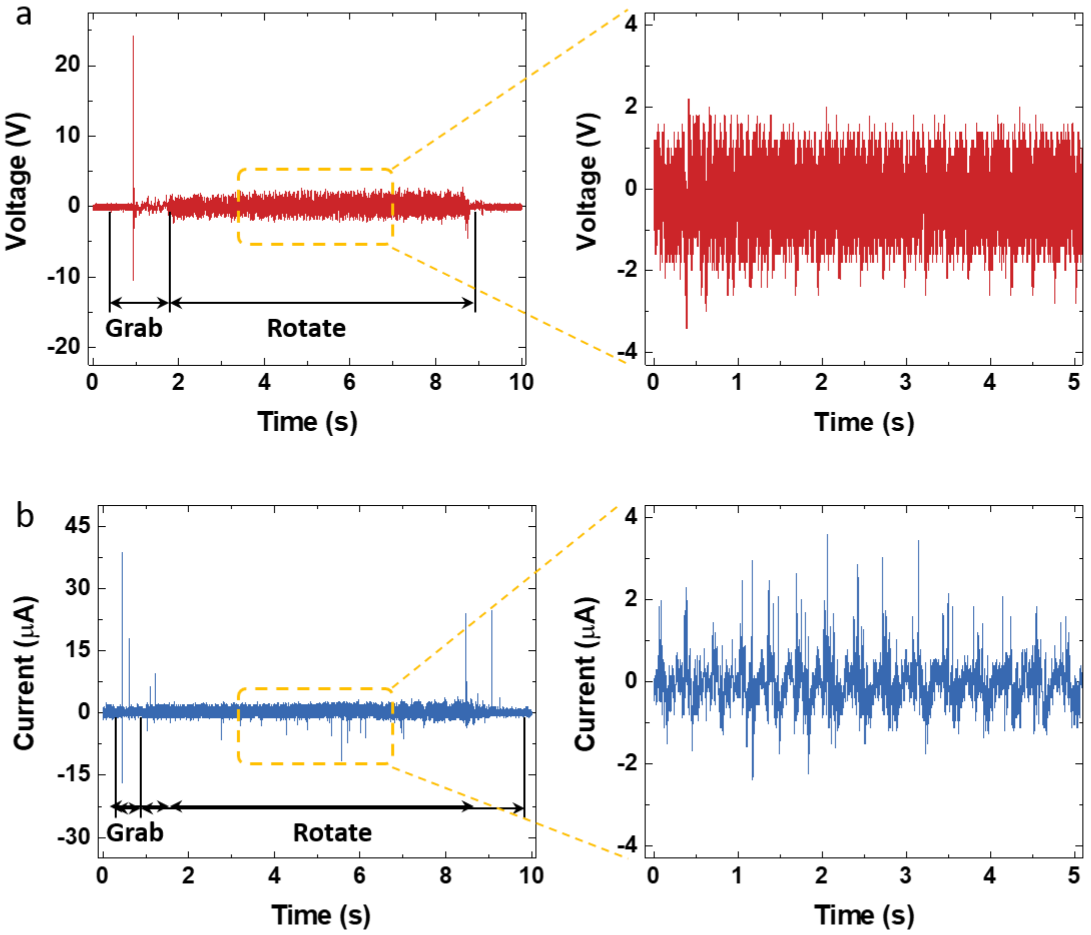

Figure S6. V_{OC} and I_{CC} output of casing generator. (a) V_{OC} output of casing generator (right) and magnified plot (left). (b) I_{CC} output of casing generator (right) and magnified plot (left).

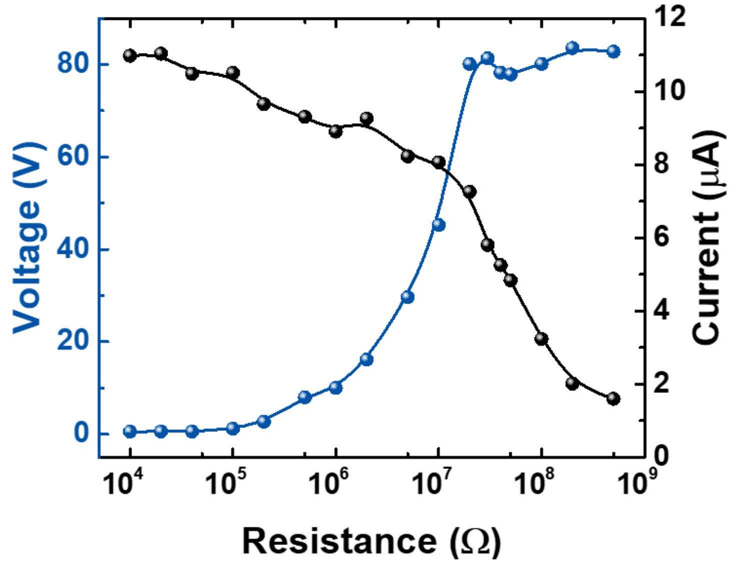

Figure S7. Voltage and current output of gyroscopic generator depending on external resistance.
